# *Salmonella* Virchow Infection of the Chicken Elicits Cellular and Humoral Systemic and Mucosal Responses, but Limited Protection to Homologous or Heterologous Re-Challenge

**DOI:** 10.3389/fvets.2014.00006

**Published:** 2014-10-09

**Authors:** Anne-Marie Salisbury, Gail Leeming, Georgios Nikolaou, Anja Kipar, Paul Wigley

**Affiliations:** ^1^Department of Infection Biology, Institute of Infection and Global Health, University of Liverpool, Neston, UK; ^2^School of Veterinary Science, University of Liverpool, Neston, UK; ^3^Vetsuisse Faculty, Institute of Veterinary Pathology, University of Zurich, Zurich, Switzerland

**Keywords:** *Salmonella* infections, animal, vaccines, humoral immune response, cellular immune response, chicken, cytokines

## Abstract

*Salmonella enterica* serovar Virchow usually causes mild gastroenteritis in humans; however, it is frequently invasive and many isolates are resistant to a broad-range of therapeutic antimicrobials. Poultry meat is considered a major source of human infection. In this study, we characterize the infection biology and immune response to *S*. Virchow in chickens and determine protection against homologous and heterologous re-challenge, with *S*. Virchow or *S*. Typhimurium. Following oral infection of 7-day-old chickens, *S*. Virchow colonized the gastrointestinal tract and the spleen. Infection elicited an increase in specific IgA, IgG, and IgM antibodies and relative quantitative changes in several leukocyte populations, including CD3, CD4, CD8α, CD8β, MHC II, KuL01, and γδ TCR positive cells, both in the gastrointestinal tract and systemically. Increased expression of pro-inflammatory cytokines IL-1β and IL-6 and the chemokine CXCLi2 was also found. Primary infection with *S*. Virchow offered limited systemic protection against re-challenge with *S*. Virchow or *S*. Typhimurium, but no protection against cecal colonization. In conclusion, *S*. Virchow exhibits similar infection biology and immune responses in the chicken to that previously described for *S*. Typhimurium. Unlike *S*. Typhimurium, *S*. Virchow infection is poorly protective to homologous and heterologous re-challenge. These findings suggest that *S*. Virchow is capable of colonizing the chicken well and therefore, presents a risk of entering the food chain in meat production. Furthermore, the development of vaccines that protect effectively against *S*. Virchow and indeed multivalent vaccines that protect across all *Salmonella* serogroups in the chicken would appear to remain a challenging proposition.

## Introduction

One of the main sources of human non-typhoidal salmonellosis is through the consumption of contaminated poultry meat and eggs (1–3). *Salmonella enterica* serovar Enteritidis (*S*. Enteritidis) and *S. enterica* serovar Typhimurium (*S*. Typhimurium) are the most common serovars causing human foodborne salmonellosis worldwide and are usually associated with mild gastroenteritis (4). Since the 1990s, *S. enterica* serovar Virchow (*S*. Virchow) has increased in prevalence in the UK and has been the third most frequent serovar isolated from human cases in recent years (5, 6). *S*. Virchow infection is most commonly associated with gastroenteritis in humans, but causes bacteremia more frequently than *S*. Typhimurium or *S*. Enteritidis, especially in immuno-compromised patients and in children (5–7). *S*. Virchow is one of the five serovars that have been given priority by the European Union (EU) for control of entry into the food chain, due to their significant risk to public health (8–10).

In Israel, *S*. Virchow has a uniquely high prevalence and association with invasive disease in humans (11, 12). In Switzerland, it has been ranked between the 4th and 8th most frequently isolated serovar between 2004 and 2009 and is a common cause of human salmonellosis in Australia and other Oceanic countries (13, 14). In other countries, including the United States, *S*. Virchow gastroenteritis in humans is less common, but cases are often associated with invasive infection (15). Increased antimicrobial resistance of *S*. Virchow has been reported in several previous studies (16–20). Recent studies on *Salmonella* prevalence associated with developing poultry industries in Bangladesh have also indicated *S*. Virchow to be a common problem (21, 22). Therefore, *S*. Virchow is a continuing and growing public health problem worldwide, being associated with invasive disease in humans and showing high antimicrobial resistance to therapeutic drugs.

Although *S*. Virchow is commonly isolated from chickens, its mechanisms of invasion and pathogenic behavior is poorly understood, as is the case with other serovars from serogroup C. A previous study has shown that intravenous infection of poultry with *S*. Virchow leads to systemic infection and colonization of organs such as the spleen, to similar levels as *S*. Enteritidis and *S*. Typhimurium (23). However, this study did not address oral infection, which is the more likely route of *Salmonella* infection in poultry, via fecal–oral transmission in the chicken house. Additionally, previous studies have shown that *S*. Virchow can survive in egg yolk, but has a limited ability to survive in albumen and on egg shells, suggesting the main vehicle of transmission to humans is poultry meat, rather than eggs (23, 24).

The aims of the current study were to characterize the infection biology of *S*. Virchow, following oral infection of chickens. Additionally, we aimed to determine the humoral, cellular, and cytokine response of the immune system following infection, to obtain information for future immunologically based preventative or therapeutic approaches, following further research. Finally, we aimed to get an indication of the protection and cross-protection offered by primary infection with *S*. Virchow against homologous and heterologous re-challenge with *S*. Virchow and *S*. Typhimurium, respectively.

## Materials and Methods

### Bacterial strains

*Salmonella* Virchow 60 was selected from a panel of 12 previously characterized *S*. Virchow isolates (25). *S*. Typhimurium F98 was included in both infection experiments for a comparison, as it is a well characterized strain in chickens (26–28). Bacterial strains were grown from glycerol stocks maintained at −70°C, in 10 ml LB broth, in an orbital shaking incubator overnight, at 37°C and 150 rpm.

### Experimental animals

All work was conducted in accordance with UK legislation governing experimental animals under project license PPL 40/3063 and was approved by the University of Liverpool ethical review process prior to the award of the license. Chicks were reared in the high-biosecurity poultry unit, University of Liverpool, in secure floor pens at a temperature of 30°C until 3 weeks of age, then at 20°C. Birds were allowed *ad libitum* access to water and vegetable protein-based laboratory poultry pelleted diet (SDS, Witham, Essex, UK). All animals were checked a minimum of twice daily to ensure their health and welfare.

### Experiment 1: immunological and pathological changes following infection

One-day-old Rhode-Island Red chicks were obtained from the Pirbright Institute, Compton, UK.

Forty-five chicks were housed separately, in 3 groups of 15 animals. At 7 days of age, Group 1 were orally challenged with 10^8^ CFU *S*. Virchow 60 in LB broth, Group 2 were orally challenged with 10^8^ CFU *S*. Typhimurium F98 in LB broth and Group 3 remained uninfected (controls). The chickens were checked twice daily, for any signs of morbidity and for any mortality. At 5, 11, and 26 days post infection (DPI), five chickens from each group were randomly selected and killed by cervical dislocation for post mortem examination. At post mortem examination, tissue samples were taken aseptically from the spleen and liver into sterile weighed containers. The cecal contents were obtained by removing the caeca aseptically, then by emptying the digesta within each cecum into a sterile container.

### Experiment 2: protection and cross-protection to re-challenge

Forty-eight 1-day-old Rhode-Island Red chicks were housed separately in 2 groups of 24 animals. At 7 days of age, the chickens in Group 1 were orally challenged with 10^8^ CFU *S*. Virchow 60 in 0.3 ml LB broth. Group 2 remained uninfected as a control. Chickens were checked twice daily, for any signs of morbidity and for any mortality. To determine intestinal clearance of *Salmonella*, cloacal swabs were taken weekly from five randomly selected chickens in each group. Swabs were directly plated onto brilliant green agar (BGA) and then enriched in selenite broth for *Salmonella* detection. BGA plates and enriched swabs were incubated overnight at 37°C and the enriched swabs were re-plated on BGA and incubated overnight at 37°C. Clearance of *Salmonella* was found at 11 weeks post infection (WPI). At 13 weeks post primary infection (WPPI), group 1 and group 2 were each divided into groups of between 10 and 12 birds. Birds were challenged or re-challenged with 10^8^ CFU *S*. Virchow (Group 1 = re-challenge, Group 3 = challenge) or *S*. Typhimurium F98 (Group 2 = re-challenge, Group 4 = challenge). At 3 and 5 days post-secondary infection, five or six chickens from each group were randomly selected and killed by cervical dislocation for post mortem examination and samples taken as described above.

### Enumeration of bacteria

During both infection experiments, cecal contents and spleens were collected during necropsy for bacteriology and diluted 1:10 in 1 × PBS. The spleen was homogenized using a MicroStomacher 80 (Seward, UK) and cecal contents were mixed using a vortex, to form a suspension. Samples were serial-diluted in 1 × PBS and plated onto BGA. Plates were incubated for 18 h at 37°C and the bacteria were enumerated. Negative samples were enriched overnight at 37°C in selenite broth and then plated onto BGA, to determine if samples contained *Salmonella*.

### Histological examination

Samples of ileum and spleen from animals in experiment 1 were fixed in 4% paraformaldehyde for 24–48 h, then trimmed and routinely paraffin wax embedded. Sections (3–5 μm) were prepared and stained with hematoxylin-eosin (HE) and independently assessed histologically by two veterinary pathologists (Georgios Nikolaou and Anja Kipar), to determine any histological changes in response to infection (29).

### Production of soluble *Salmonella* lysate antigen

Soluble *Salmonella* lysate antigen for each serovar was prepared as described previously (28). Overnight cultures of *S*. Virchow 60 and *S*. Typhimurium F98, as described above, were used to inoculate 100 ml LB broth, which was then incubated overnight at 37°C and 150 rpm. Cultures were aseptically poured into sterile tubes and centrifuged at 4080 × *g* for 25 min at 4°C to obtain bacterial pellets. The supernatant was poured off and the bacterial pellet was suspended in 20 ml 1 × PBS. Bacterial suspensions were incubated in a waterbath at 65°C for 5 h and a small aliquot was plated onto nutrient agar and incubated at 37°C overnight, to confirm no viable *Salmonella* remained. Following this, bacterial suspensions were sonicated in 10 ml volumes in 20 s bursts on ice at an amplitude of 15 μm, using a soniprep 150 (MSE Scientific Instruments, UK), for a total of 10 times, allowing the suspension to cool for 1 min between each burst. Suspensions were centrifuged at 4080 × *g* for 20 min at 4°C and then centrifuged at 30000 × *g* for 20 min at 4°C. Protein concentrations were measured using the Bradford protein determination kit (Merck, Poole, UK). The soluble antigen preparations were stored in aliquots at −20°C.

### Enzyme-linked immunosorbent assay

Serum was obtained by removing blood from the heart at necropsy, allowing it to clot, then removing the serum after centrifuging at 13000 × *g* for 5 min for. Serum samples were then stored as aliquots at −20°C until used. Levels of serum specific antibodies against *S*. Virchow and *S*. Typhimurium soluble antigen were determined at each time point, as described previously (28, 30). Each sample was run in triplicate. Flat-bottomed 96-well plates were coated with 100 μl/well of *S*. Virchow or *S*. Typhimurium soluble antigen, diluted in carbonate–bicarbonate buffer (pH 9.6) to a concentration of 16.2 μg/ml and incubated overnight at 4°C. Subsequently, the plates were washed three times with PBS Tween-20 (0.05%). They were then incubated with 3% blocking buffer (consisting of 0.05% Tween-20 in PBS and 3% skimmed milk powder) for 1 h at 37°C and washed with PBS Tween-20 (0.05%). Serum samples were diluted in blocking buffer for the detection of IgA (1:25), IgM (1:400), and IgG (1:400). Plates were incubated with the diluted chicken serum for 1 h at 37°C and washed three times in PBS Tween-20 (0.05%). Specific antibodies were detected by incubating the samples with alkaline phosphatase conjugated to either goat anti-chicken IgA (1:20000), IgM (1:1000), or IgG (1:2000) (Serotec, Oxford, UK) diluted in blocking buffer, for 1 h at 37°C. Plates were washed with PBS Tween-20 (0.05%) and incubated with 100 μl per well of *p*-nitrophenyl phosphate in the dark for 30 min at room temperature. The reaction was stopped by addition of 100 μl 3N sodium hydroxide to each well. Absorbance was determined using a microplate reader at 405 nm. Negative control serum was obtained from *Salmonella*-free animals and positive controls for *S*. Typhimurium were included using positive serum from previous studies. No positive control was available for *S*. Virchow at the start of the experiment, but a positive sample from the first ELISA run was included on subsequent plates as a control to ensure there was little variation from plate-to-plate.

### Immunohistochemistry

Spleen, ileum, and cecal tonsil were collected from all animals of experiment 1 and snap frozen in liquid nitrogen on cork plates in OCT (Tissue-tek, UK). Serial sections (10 μM) were cut from each sample, placed on poly-l-lysine coated slides (VWR International, UK) and fixed in acetone for 10 min. Tissue sections were incubated overnight at 4°C with monoclonal antibodies against chicken CD3, CD4, CD8α, CD8β, MHC II (B cells, antigen presenting cells, macrophages, and monocytes) (31), KuL01 (monocytes, macrophages, interdigitating cells, and activated microglia cells) (31), γδ TCR and Bu1a (B cells and subsets of monocytes and macrophages), antigens (Southern Biotechnology, Cambridge, UK), and diluted 1:100 in 1 × TBS (tris buffered saline). A Vectastain Elite ABC kit (Vector Laboratories, Peterborough, UK) was used for the detection of antibody binding. Sections were incubated with the secondary antibody (biotinylated horse anti-mouse, diluted 1:100 in 1 × TBS) for 30 min at room temperature. The reaction was visualized by incubating the slides with 3′3-diaminobenzidine, followed by counterstaining with Papanicolaou’s hematoxylin and mounting with DPX mounting media (VWR International).

Immunocytochemically stained sections were analyzed using a Nikon Eclipse 80i microscope and NIS-elements BR 3 software. From each sample, five high power fields (400×) of the spleen and cecal tonsil were analyzed. The first field was from the center of the tissue, followed by four fields around the central field. Cells expressing the respective antigen were manually counted for all five fields and then an average cell count was determined for each chicken. A different approach was taken for the Bu1a^+^ cells as these cells comprised the lymphatic follicles in the cecal tonsil. The area of every stained follicle present on the tissue was measured to determine if the follicles changed size over time, after infection. An average follicle area was calculated for each chicken. No differences were found in the intensity of the inflammatory infiltrate between the villous and crypt lamina propria; therefore, cell counts were determined in the villi. Ten medium power (200×) fields were analyzed per chicken; fields were selected in which longitudinal sections of villi occupied the whole diameter of the field. An average cell count from 10 fields was determined for each chicken.

### 2^−ΔΔCT^ real-time RT-PCR for cytokine expression

From animals of experiment 1, spleen and cecal tonsil were collected after euthanasia at 5, 11, and 26 DPI and stored in RNAlater (Sigma-Aldrich, UK) at −20°C. Total RNA was prepared from the tissue samples using an RNeasy kit and following the manufacturer’s instructions (Qiagen, UK). The transcription levels of cytokines IL-1β, IL-6, IL-4, IFN-γ, and the chemokine CXCLi2 were determined using Rotor-Gene Q software v.4.14.2 (Qiagen, UK). Primers and probes for the selected cytokines and chemokine have been described previously (32, 33). One-Step RT-PCR was performed using the Rotor-Gene Probe RT-PCR Master Mix (Qiagen; includes RT stage) in a final concentration of 1 × 0.25 μl Rotor-Gene RT mix, 0.8 μM of both the forward and reverse primers, 0.2 μM of the probe, and 1 μl RNA made up to a total volume of 25 μl with RNase-free water. The following cycling conditions were used for amplification: 50°C for 10 min, 95°C for 5 min, followed by 40 cycles of 95°C for 5 s and 60°C for 10 s.

Each sample was run in triplicate and an average C_T_ value was taken for each group. The threshold for C_T_ values was set between 0.20 and 0.23. C_T_ values were normalized firstly to the endogenous control and then to the uninfected control group (34). Expression levels in the infected groups were represented as the fold-change in expression compared to the uninfected control.

### Statistical analysis

Statistical analysis was performed using SPSS version 20.0 software. Bacterial counts, immunohistochemical cell counts, and RT-PCR C_T_ values were compared using one-way ANOVA. Significance between the values was taken if the *P* value was <0.05.

## Results

### Bacteriology

#### Experiment 1

**Figure 1:**
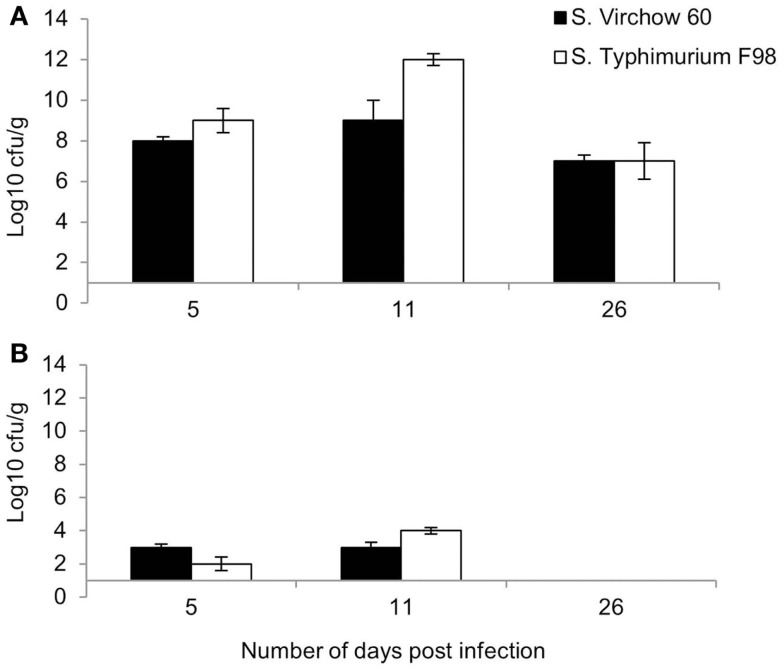
**Mean viable counts of *S*. Virchow and *S*. Typhimurium in the cecal contents (A) and spleen (B) at 5, 11, and 26 DPI, following infection of 7-day old chickens**. Data shown are based on five birds per group at each time point. Error bars represent the standard error of the mean.

Following primary infection, *S*. Virchow 60 and *S*. Typhimurium F98 were found in the cecal contents at log_10_ 8.0 CFU/g and log_10_ 9.0 CFU/g at 5 DPI (Figure 1A). Bacterial counts in the caeca peaked at 11 DPI, reaching levels of up to log_10_ 9.0 CFU/g and log_10_ 12.0 CFU/g for *S*. Virchow and *S*. Typhimurium F98, respectively. By 26 DPI, colony counts had begun to decline in both infected groups.

Both *S*. Virchow 60 and *S*. Typhimurium F98 could be detected in the spleen by 5 DPI, at log_10_ 3.0 CFU/g and log_10_ 2.0 CFU/g, respectively (Figure 1B). Counts peaked at 11 DPI; however, no *Salmonella* could be detected in the spleen of either infected group by 26 DPI.

#### Experiment 2

**Figure 2:**
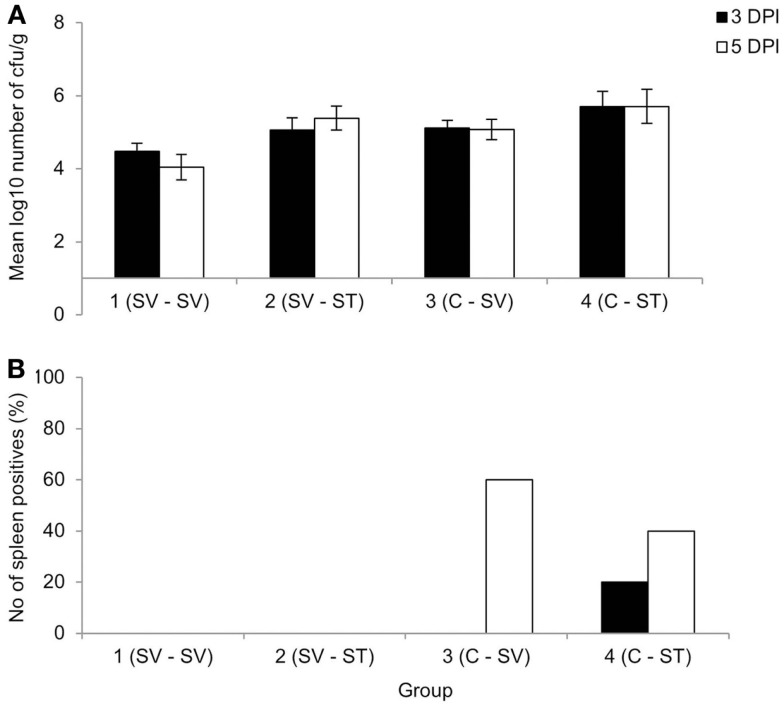
**Mean viable counts of bacteria in the cecal contents (A) and percentage of spleen positives following enrichment (B) at three and five DPI, following re-challenge after primary *S*. Virchow infection**. Data shown are based on five birds per group at each time point. Group 1: primary infection with *S*. Virchow, secondary infection with *S*. Virchow; Group 2: primary infection with *S*. Virchow, secondary infection with *S*. Typhimurium; Group 3: primary infection with *S*. Virchow; and Group 4: primary infection with *S*. Typhimurium. Error bars represent the standard error of the mean.

No animals were lost as a result of infection, though three were euthanized due to unrelated welfare issues. Clearance after primary *S*. Virchow infection occurred by 11 WPPI. At 13 WPPI, Group 1 was divided and given a homologous or heterologous re-challenge and Group 2 was divided and infected with *S*. Virchow 60 or *S*. Typhimurium F98 for age-matched controls. At 3 and 5 days post challenge, the cecal content counts were lower in the re-challenged groups compared to the age-matched control groups (Figure 2A), although the difference in bacterial count was not significant (*P* > 0.607). Viable *Salmonella* could not be directly isolated from the spleen from any of the groups at either time point. Therefore, spleen samples were enriched in selenite broth and the percentage of positive and negative spleens for each group was determined. The re-challenged groups were negative following enrichment, whereas at 3 days post challenge 20% of the *S*. Typhimurium age-matched control group were positive and at 5 days post challenge 40 and 60% of the *S*. Virchow and *S*. Typhimurium age-matched control groups were positive, respectively (Figure 2B).

### Histopathology

The histological examination of ileum and spleen from animals in experiment 1, i.e., at 5, 11, and 26 DPI was undertaken to determine the type and degree of pathological changes in response to *S*. Virchow infection. In most control animals, the ileum exhibited scattered lymphocytes in the lamina propria mucose and occasional lymphocyte exocytosis into the lamina epithelialis mucose. In some animals, the mucosal infiltration was more prominent (mild to moderate) and comprised not only lymphocytes, but also macrophages and scattered heterophils. At 5 and 11 DPI, chickens infected with either *Salmonella* strain exhibited a slight increase in lymphocytes in the lamina propria, but the lamina epithelialis generally remained unaltered. Animals infected with *S*. Virchow also exhibited occasional loose aggregates of heterophils in the lamina propria mucose in particular in the villi.

Spleens exhibited a white pulp composed of small to moderately sized lymphatic follicles without obvious germinal center reaction, and inconspicuous T cell zones in all groups. The red pulp was moderately cellular and contained some heterophils in a few control animals and generally a low to moderate number of heterophils in chickens infected with either *Salmonella* strain at 5 and 11 DPI. This difference was not apparent by 26 DPI.

### Humoral immune response

#### Experiment 1

**Figure 3:**
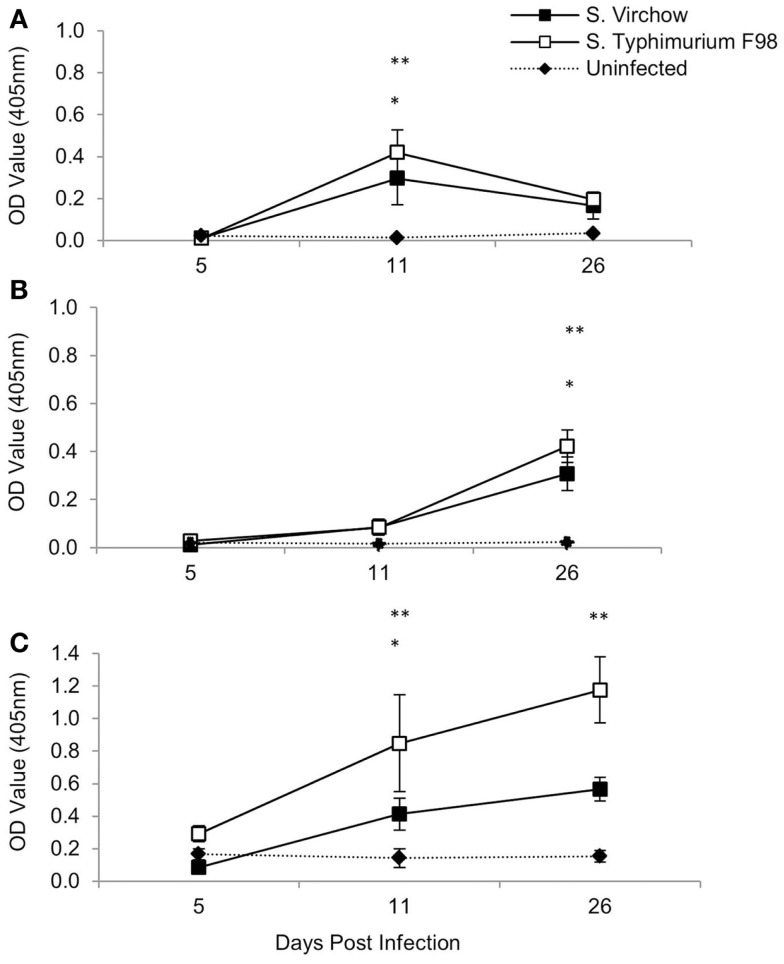
**Antigen-specific serum IgM (A), IgA (B), and IgG (C) against *S*. Virchow and *S*. Typhimurium at 5, 11, and 26 DPI, following infection of 7-day old chickens**. Data shown are based on five birds per group at each time point. Error bars represent the standard error of the mean. An asterisk indicates that there was a significant difference between the *S*. Virchow infected group and uninfected group (*) or the *S*. Typhimurium infected group and the uninfected group (**) (*P* < 0.05).

Specific IgM, IgG, and IgA antibodies were detected following infection with both *S*. Virchow 60 and *S*. Typhimurium F98 (Figure 3). Between 5 and 11 DPI, serum IgM levels against *S*. Virchow increased rapidly, peaking at 11 DPI. By 26 DPI, anti-*Salmonella* IgM had declined toward levels found in the uninfected group. IgA and IgG increased more slowly, but to greater levels than IgM. Between 5 and 11 DPI, IgA levels against *S*. Virchow increased slowly and were not considerably higher than in the uninfected group. However, between 11 and 26 DPI, there was a sharp increase in IgA and by 26 DPI, levels were considerably higher than in the uninfected group. IgG levels against *S*. Virchow increased steadily throughout the infection period. The IgA, IgM, and IgG response against *S*. Virchow followed a pattern similar to that seen against *S*. Typhimurium F98 throughout the experiment.

#### Experiment 2

**Figure 4:**
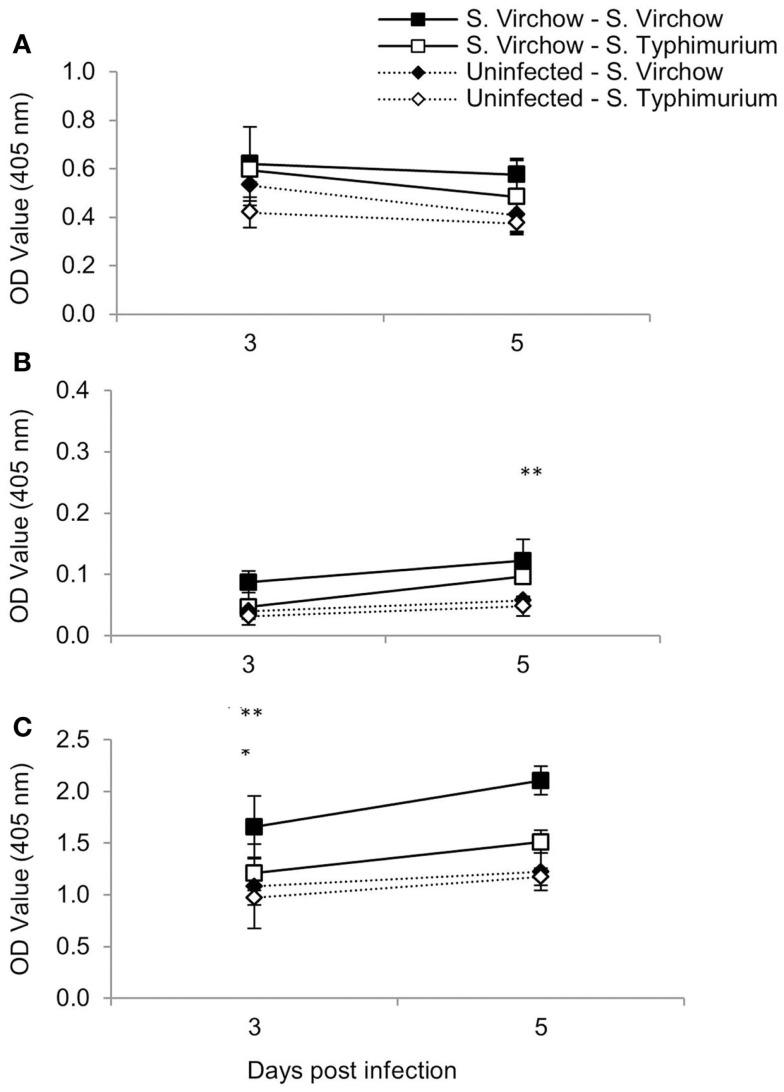
**Antigen-specific serum IgM (A), IgA (B), and IgG (C) following primary and secondary infection of chickens with *S*. Virchow and *S*. Typhimurium**. Data shown are based on five birds per group at each time point. Error bars represent the standard error of the mean. An asterisk indicates that there was a significant difference between group 1 and group 2 (*) or group 3 and group 4 (**) (*P* < 0.05).

At three and five DPI, serum samples were collected to determine the specific IgA, IgG, and IgM production against *S*. Virchow 60 and *S*. Typhimurium F98 after re-challenge, compared to age-matched controls (Figure 4). Serum IgA antibody levels increased in all four groups between 3 and 5 days post challenge, although they were considerably higher in the re-challenged groups compared to the age-matched controls. Serum IgG antibody levels increased in all four groups between 3 and 5 days post challenge; however, they were highest in the *S*. Virchow re-challenged group at both time points. By 5 days post challenge, IgG levels were higher in the re-challenged groups compared to the age-matched controls. Serum IgM antibody levels decreased between 3 and 5 days post challenge in all four groups. IgM titers were similar between all four groups, although the highest IgM levels were found in the *S*. Virchow re-challenged group, followed by the *S*. Typhimurium re-challenged group.

### Cellular responses

**Figure 5:**
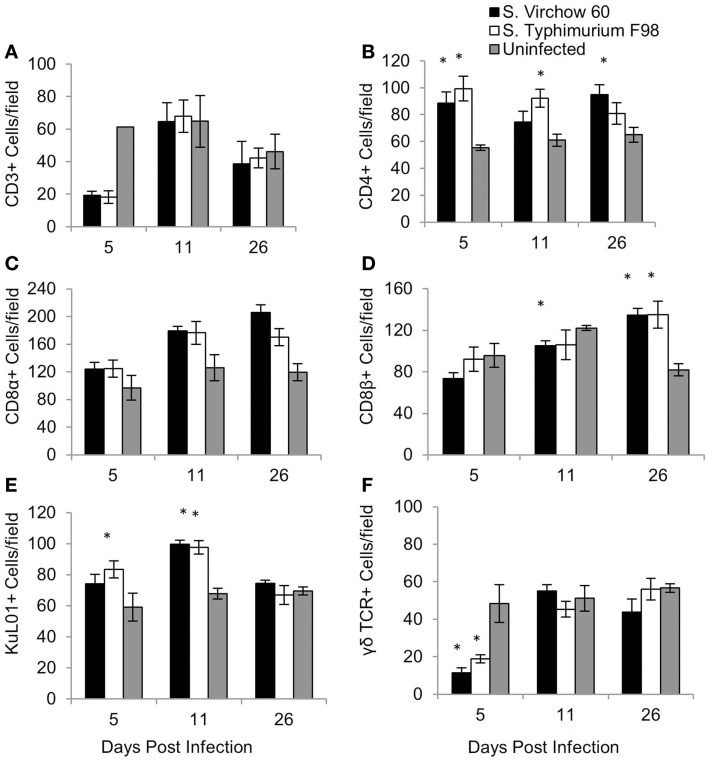
**Average number of CD3 (A), CD4 (B), CD8α (C), CD8β (D), KuL01 (E), and γδ TCR (F) positive cells/field in the spleen of chickens during *S*. Virchow and *S*. Typhimurium infection compared to in uninfected chickens**. Data shown are based on minimum of five birds per group at each time point (*n* = 5 or 6) and five fields of view per bird. Error bars represent the standard error of the mean. The asterisk (*) indicates a significant difference between the infected group and uninfected group.

**Figure 6:**
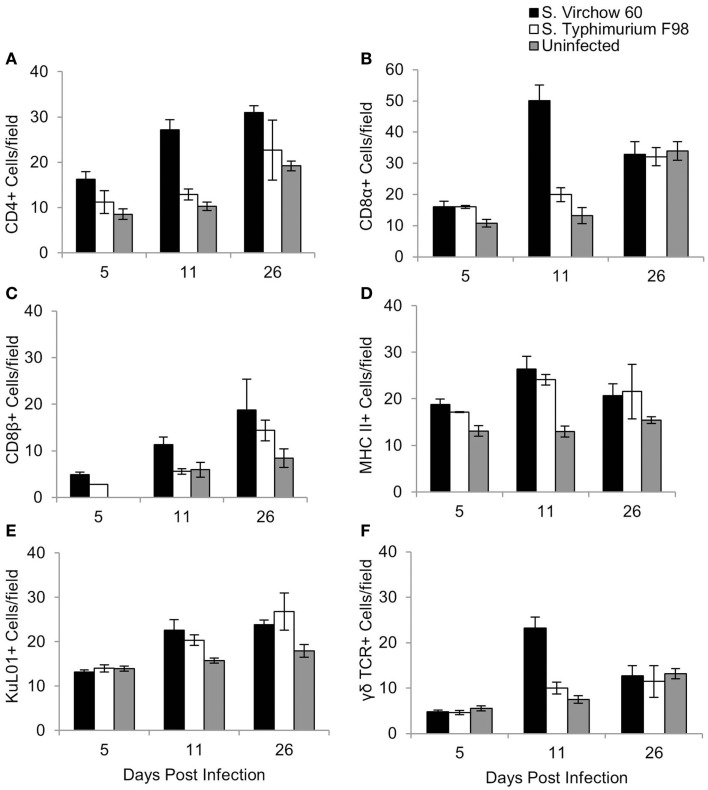
**Average number of CD4 (A), CD8α (B), CD8β (C), MHC II (D), KuL01 (E), and γδ TCR (F) positive cells/field in the ileum of chickens during *S*. Virchow and *S*. Typhimurium infection compared to in uninfected chickens**. Data shown are based on minimum of 5 birds per group at each time point (*n* = 5 or 6) and 10 fields of view per bird. Error bars represent the standard error of the mean. The asterisk (*) indicates a significant difference between the infected group and uninfected group.

**Figure 7:**
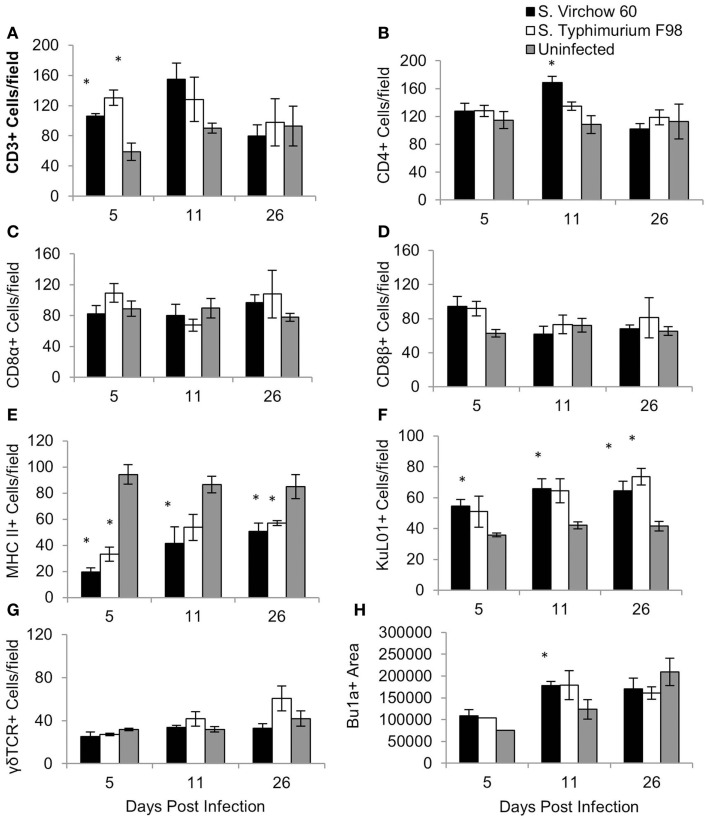
**Average number of CD3 (A), CD4 (B), CD8α (C), CD8β (D), MHC II (E), KuL01 (F), γδ TCR (G), and Bu1a (H) positive cells/field in the cecal tonsil of chickens during *S*. Virchow and *S*. Typhimurium infection compared to in uninfected chickens**. Data shown are based on minimum of five birds per group at each time point (*n* = 5 or 6) and five fields of view per bird. Error bars represent the standard error of the mean. The asterisk (*) indicates a significant difference between the infected group and uninfected group.

To quantify changes in the cellular immune response following *S*. Virchow infection, spleen (Figure 5), ileum (Figure 6), and cecal tonsil (Figure 7) specimens were collected from animals in experiment 1, euthanized at 5, 11, and 26 DPI. For the spleen, five high power fields (40X) were selected and the positively stained cells were manually counted. Immunocytochemistry showed that at five DPI, numbers of CD4^+^, CD8α^+^, and KuL01^+^ cells were increased in the spleen in chickens infected with *S*. Virchow, compared to those in uninfected chickens, with the increase in CD4^+^ cells being significant (*P* < 0.05). At 11 DPI, CD4^+^, CD8α^+^, and KuL01^+^ cells had increased further in the *S*. Virchow infected group, to significantly higher amounts than those found in the uninfected group (*P* < 0.003). By 26 DPI, CD8β^+^ cells had significantly increased in the *S*. Virchow infected group above the numbers found in the uninfected group (*P* < 0.0025). In contrast, CD3^+^ and γδ TCR^+^ cells decreased in the spleen of *S*. Virchow infected chickens, compared to the uninfected chickens at 5 DPI; however, they had returned to levels similar to those found in the uninfected group by 11 DPI.

For the ileum, 10 medium power fields (×200) were selected and the cells were manually counted. An increase in CD4^+^, CD8α^+^, CD8β^+^, and MHC II^+^ cells was observed at five DPI in the *S*. Virchow infected group, in comparison to the uninfected group, with the increase in CD4^+^ and MHC II^+^ cells being significant (*P* < 0.028). CD4^+^, CD8α^+^, CD8β^+^, and MHC II^+^ cells increased further in the *S*. Virchow infected group at 11 DPI, when a significant increase in KuL01^+^ and γδ TCR^+^ cells was also found (*P* < 0.016). At 26 DPI, CD4^+^, MHC II^+^, CD8β^+^, and KuL01^+^ cells were still elevated in the *S*. Virchow infected group; however, CD8α^+^ and γδ TCR^+^ cells had returned to levels similar to those seen in the uninfected group. In the ileum, the number of several cell populations was higher in the *S*. Virchow infected group compared to the *S*. Typhimurium infected group and the uninfected group. Although the number of CD4^+^ cells were increased in both infected groups at each time point, they were considerably higher in the *S*. Virchow infected group. CD8α^+^ and CD8β^+^ cells were increased in the infected groups throughout the infection experiment; however, the cell counts were significantly higher, for both subpopulations, in the *S*. Virchow infected group compared to the *S*. Typhimurium infected group and the uninfected group at 11 DPI (*P* < 0.036). MHC II^+^ cells were significantly more numerous in the infected groups at 5 (*P* < 0.028) and 11 (*P* < 0.011) DPI and numbers remained higher than those of the uninfected group at 26 DPI. At 11 DPI, a significant increase was seen in KuL01^+^ (*P* < 0.016) and γδ TCR^+^ (*P* < 0.001) cells in both of the infected groups and was still seen at 26 DPI.

For the cecal tonsil, five high power fields (×400) were selected and the positively stained cells were counted manually. The quantity of CD3^+^, CD8β^+^, and KuL01^+^ cells had increased in the *S*. Virchow infected group by five DPI, with the number of CD3^+^ and KuL01^+^ cells being significantly higher than in the uninfected group (*P* < 0.016). At 11 DPI, CD3^+^, KuL01^+^ cell numbers had increased further in the *S*. Virchow infected group. A significant increase was also found in CD4^+^ cells (*P* < 0.05). At 26 DPI, KuL01^+^ cells were still significantly higher in the *S*. Virchow infected group compared to the uninfected group. Throughout the infection period MHC II^+^ cells were significantly lower in the *S*. Virchow infected group compared to the uninfected group (*P* < 0.045). The size of the Bu1a^+^ follicles had increased in the *S*. Virchow infected group by 5 DPI and had increased further at 11 DPI (*P* < 0.036). Alterations in the cell numbers in the *S*. Virchow infected group were similar to those in the *S*. Typhimurium F98 infected group, throughout the infection period.

### Cytokine and chemokine expression

**Figure 8:**
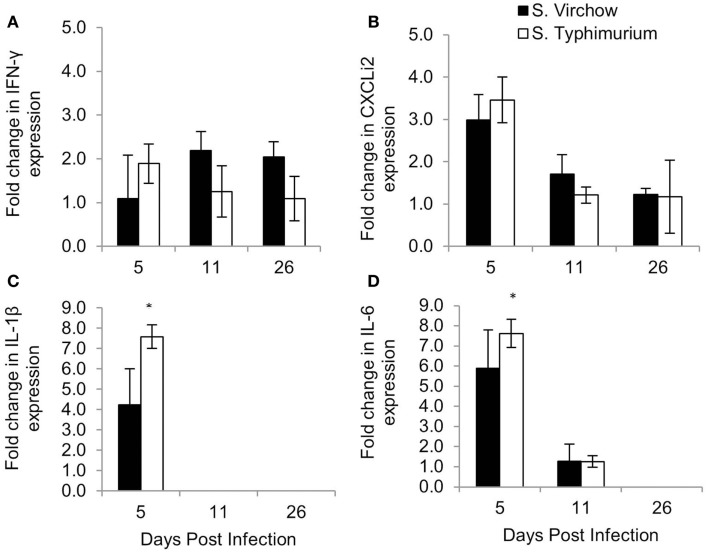
**Relative expression of IFN-γ (A), CXCLi2 (B), IL-1β (C), and IL-6 (D) in the spleen of chickens infected with *S*. Virchow and *S*. Typhimurium F98 compared to uninfected chickens, at 5, 11, and 26 DPI**. Data shown are based on minimum of five birds per group at each time point (*n* = 5 or 6). Error bars represent standard error of the mean. An asterisk (*) indicates that there was a significant fold-change between the infected group and the uninfected group.

**Figure 9:**
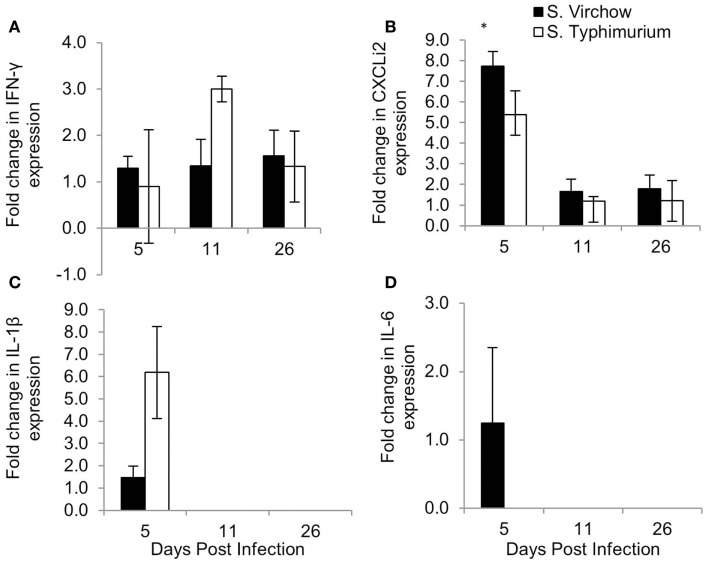
**Relative expression of IFN-γ (A), CXCLi2 (B), IL-1β (C), and IL-6 (D) in the cecal tonsil of chickens infected with *S*. Virchow and *S*. Typhimurium F98 compared to uninfected chickens, at 5, 11, and 26 DPI**. Data shown are based on minimum of five birds per group at each time point (*n* = 5 or 6). Error bars represent standard error of the mean. An asterisk (*) indicates that there was a significant fold-change between the infected group and the uninfected group.

The change in expression of key cytokines and chemokines was determined in the spleen (Figure 8) and cecal tonsil (Figure 9), at each time point, throughout Experiment 1. The magnitude of the response sometimes varied within groups, which can be seen by the standard error bars. These findings have been reported previously (28).

In the spleen, IL-1β, IL-6, and CXCLi2 mRNA levels were increased in the *S*. Virchow infected group above those of the uninfected group, by 4.23, 5.90, and 2.99-fold, respectively, at five DPI. They were also increased in the *S*. Typhimurium F98 infected group above those of the uninfected group, by 7.58, 7.62, and 3.46-fold, respectively, with the increase in IL-1β and IL-6 being significant. At 11 DPI, IL-1β, IL-6, and CXCLi2 transcription in the infected groups had returned to levels similar to those seen in the uninfected group. IFN-γ levels were increased in spleens of the *S*. Virchow infected group at 11 DPI by 2.19-fold. IL-4 was not expressed in the spleen at any time point during the infection period.

In the cecal tonsil, the IL-1β mRNA level was increased by 1.46-fold in the *S*. Virchow infected group at five DPI. IL-1β transcription was variable in the *S*. Typhimurium infected group, with two chickens showing a 6.19-fold increase and three chickens showing no change at five DPI. A 1.25-fold increase in the IL-6 mRNA level was found in 3/5 *S*. Virchow infected chickens at five DPI; however, IL-6 expression in the *S*. Typhimurium infected group did not change in comparison to the uninfected group. CXCLi2 expression was higher in the *S*. Virchow infected group than the uninfected group at each time point and had increased 7.73, 1.65, and 1.80-fold at 5, 11, and 26 DPI, respectively. The increase in CXCLi2 in the *S*. Virchow infected group compared to the uninfected group was significant at five DPI. CXCLi2 transcription had increased by an average of 5.39-fold in two birds from the *S*. Typhimurium F98 infected group at five DPI. IFN-γ levels increased slightly in the *S*. Virchow infected group by 1.29, 1.34, and 1.56-fold at 5, 11, and 26 DPI, respectively. In the *S*. Typhimurium infected group, IFN-γ expression was similar to the uninfected group at five DPI; however, it had increased by threefold at 11 DPI. At 26 DPI, IFN-γ expression had decreased and was 1.33-fold higher than in the uninfected group. IL-4 expression was not detected in the cecal tonsil for the duration of the experiment.

## Discussion

Previously, we demonstrated that *S*. Virchow can colonize the gastrointestinal tract and the spleen of chickens, to levels similar to those seen with *S*. Typhimurium at three DPI, suggesting *S*. Virchow may mirror the infection biology found with broad-range serovars (25). Here, we have further characterized the *S*. Virchow colonization of poultry and confirm that it exhibits an infection biology similar to *S*. Typhimurium, over an infection period of 26 days. *S*. Virchow colonized the gastrointestinal tract and the spleen by 5 DPI, with bacterial counts peaking in the gut and systemically at 11 DPI. By 26 DPI, *S*. Virchow had been cleared from the spleen, showing that it causes transient systemic infection; however, bacterial counts were still high in the cecal contents. As with *S*. Typhimurium F98, chickens infected with *S*. Virchow showed no signs of clinical illness and no significant pathological changes in the ileum during the infection period, suggesting its low pathogenicity in this host. Although, both *S*. Virchow and *S*. Typhimurium have been shown to be invasive, depending on the strain and the host they are infecting (35–39).

The histological analysis of the ileum and spleen did not reveal marked pathological changes at 5, 11, and 26 DPI and no distinct evidence of epithelial damage in the ileum. A slight increase in lymphocyte infiltration and some heterophil recruitment was seen in the ileum together with evidence of systemic heterophil release, based on the general mild increase in heterophils in the splenic red pulp at the two earlier time points. Our findings differ from those of a previous study, which examined the effect of *S*. Typhimurium in the intestine of 40-day old white leghorn chickens up to four DPI and observed evidence of epithelial damage and a more pronounced mononuclear infiltration (40). The author also found large numbers of heterophils in the intestinal lumen. However, only small numbers of heterophils were seen in the lamina propria that would be more consistent with our findings on day 5. It can also not be excluded that age and breed play a role in the response to infection. The fact that we observed evidence of systemic and local heterophil recruitment after infection is consistent with a more recent study indicating the relevance of heterophils in the innate response to bacterial, including *Salmonella*, infection in chickens (41, 42).

To our knowledge, this is the first detailed study of the humoral, cellular, and cytokine response produced by chickens in response to oral infection with *S*. Virchow. The results show that *S*. Virchow stimulates an immune response in chickens similar to that seen by other broad-range serovars. This suggests that serogroup has limited influence on innate or adaptive immune responses beyond the change in specificity of response to LPS. Indeed recent determination of the innate response to another serogroup C serovar, *S*. Infantis, supports the notion that the immunobiology of infection in the chicken is similar with all invasive broad host range serovars (43, 44). After *S*. Virchow infection, numbers of CD4^+^, CD8α^+^, and CD8β^+^ cells had increased in the ileum by five DPI, indicating a T helper and a cytotoxic T cell response had occurred. Previous studies have found variation in T cell influx into the gut, depending on location in the gastrointestinal tract, infection dose, age of the host at the time of infection, and the genetic background of the host (45–48).

MHC II^+^ cells increased in number in the ileum throughout the experiment and correlated with an increase in KuL01^+^ cells, suggesting local recruitment of macrophages and the likely role of antigen presenting cells in the control of *S*. Virchow infection. At 11 DPI, the quantity of γδ TCR^+^ cells increased in the ileum in both infected groups; however, the number of cells in the *S*. Virchow infected group was significantly higher than in the *S*. Typhimurium infected group and the uninfected group (*P* = < 0.001). An early increase in γδ TCR^+^ cells in the cecum has been shown previously, following infection of chicks with *S*. Typhimurium, *S*. Enteritidis, and *S. enterica* serovar Hadar (*S*. Hadar) (47, 49). The difference between time points during this study and previous studies may be due to different areas of the gut being sampled.

The changes in the proportion of leukocyte subpopulations were less prolonged in the cecal tonsil, compared to the ileum. CD3^+^ and CD8β^+^ cell numbers increased early in the infected groups, by 5 DPI, followed by an increase in CD4^+^ cells at 11 DPI and CD8α^+^ cells by 26 DPI. An increase of CD4^+^ cells in the cecal tonsil, following *S*. Enteritidis infection, has been previously associated with immunoglobulin class switching (50). This mechanism could explain why, in this study, CD4^+^ cells were only up-regulated in the cecal tonsil at one time point. The size of the lymphatic follicles which were comprised of Bu1a^+^ B cells in the cecal tonsil was increased in the infected groups compared to the uninfected group at 5 and 11 DPI, indicating the humoral immune response has a role in clearance of *Salmonella* infection. Throughout the infection experiment, MHC II^+^ cells decreased, whereas KuL01^+^ cells increased, in the cecal tonsil. An increase in KuL01^+^ cells in the cecal tonsil following *S*. Enteritidis infection has been shown previously (43) and could indicate antigen presentation to immune cells in the cecal tonsil.

CD4^+^ and CD8α^+^ cell numbers increased in the spleen in infected groups and remained elevated for the duration of the experiment. CD8β^+^ cells did not increase in the spleen until 26 DPI in the infected groups. Varying results have been found for these subpopulation changes in the spleen and could be a result of age of the chickens when infected, infecting serovar or dose (45, 50, 51). At five DPI, the number of CD3^+^ and γδ TCR^+^ cells (T cells) was decreased in the spleen in the infected groups. A decrease of lymphocytes in the spleen has been shown to coincide with an increase in the cecum following *S*. Enteritidis infection (45) and could indicate cell-trafficking from the spleen to the gut.

Changes in IFN-γ, CXCLi2, IL-1β, and IL-6 transcription were found in the spleen and cecal tonsil during *S*. Virchow and *S*. Typhimurium infection; although, the magnitude of the response varied within groups. This was particularly evident in the cecal tonsil, as some chickens would exhibit a response, whereas others would not. Variability in cytokine and chemokine response in the cecal tonsil within the same group has been shown in previous studies and may be due to differences in immunological maturation from chicken to chicken (28, 50).

An increase in IL-1β, IL-6, and CXCLi2 in the spleen and cecal tonsil of chickens occurred by 5 DPI, in both infected groups, showing that a rapid inflammatory response occurs against *S*. Virchow, in a similar manner to *S*. Typhimurium. IL-6 remained elevated in the spleen until 26 DPI, which has previously been associated with lymphocyte and macrophage development, rather than an acute inflammatory response (30). CXCLi2 is a pro-inflammatory chemokine that is highly homologous to human IL-8 and is important for early immune responses in the gut, including an influx of heterophils (52–54). The increase in IL-1β, IL-6, and CXCLi2 transcription in the spleen and cecal tonsils shows that like *S*. Typhimurium and other broad-range serovars, *S*. Virchow elicits a strong immune response in the chicken, causing a rapid inflammatory response upon infection. The response elicited is unlike that seen with host-restricted serovars, such as *S. enterica* serovar Pullorum (*S*. Pullorum) and *S. enterica* serovar Gallinarum (*S*. Gallinarum), which do not induce inflammatory responses and rely on “stealth” to invade and cause systemic or typhoidal-like disease (55).

IFN-γ expression was modestly increased in both infected groups compared to the uninfected group at every time point, in both the spleen and cecal tonsil. IFN-γ enhances the oxidative burst in macrophages against *Salmonella* infection (56). Elevated levels of IFN-γ support the idea that *S*. Virchow clearance is dependent on IFN-γ T cell mediated responses. The increased level of IFN-γ, in combination with the lack of IL-4 expression (Th2 cytokine) (57), suggests *S*. Virchow clearance is, like S. Typhimurium, primarily Th1-mediated.

Serum humoral responses showed a classical pattern of a rapid rise in IgM, followed by a rise in IgG and IgA, against *S*. Virchow infection. The humoral immune response was slightly stronger against *S*. Typhimurium than *S*. Virchow throughout the infection experiment, although it did follow the same pattern in both infected groups. Increased serum antibodies and increased amounts of B cells (Bu1a^+^) in the cecal tonsil suggest the humoral response has a role in *S*. Virchow clearance, although previous studies have shown it is not needed for *Salmonella* clearance (58, 59).

The bacteriology results from Experiment 2 showed primary infection with *S*. Virchow offers some protection against systemic spread, following secondary infection, but no significant reduction in cecal colonization. Additionally, primary infection with *S*. Virchow offered cross-serogroup protection against systemic spread, following secondary infection with *S*. Typhimurium, but no protection against colonization of the gut.

*Salmonella*-specific IgA, IgG, and IgM were detected following challenge in all four infected groups but levels were always highest in the *S*. Virchow homologous re-challenge group (Group 1). These results are indicative of an antigen-specific secondary immune response in previously challenged birds. Additionally, the quicker and greater antibody response observed in the *S*. Typhimurium heterologous re-challenge group (Group 2) compared to the age-matched *S*. Typhimurium infected group (Group 4) indicates a degree of immunological cross-reactivity against *S*. Virchow and *S*. Typhimurium, though little protection.

A previous study, investigating the cross-protection and cross-reactivity against *S*. Typhimurium and *S*. Enteritidis infection found reduced cecal content and splenic bacterial counts following re-challenge, compared to the age-matched controls (60). Cross-protection offered by primary infection with *S*. Typhimurium against secondary infection with *S*. Enteritidis was more effective than vice versa (60). In addition to this study, an earlier one looked at the degree of cross-protection in mice, focusing on the protection conferred by the main LPS O antigen. Primary infection with *S*. Typhimurium or *S*. Enteritidis gave high protection against homologous re-challenge; however, no protection against challenge with the heterologous serotype. Here, we show some evidence of cross-protection elicited by *S*. Virchow against systemic infection, following heterologous re-challenge with *S*. Typhimurium, but no cross-protection against cecal colonization. It is possible that *S*. Typhimurium may have protected more effectively against secondary infection with *S*. Virchow, but our findings suggest there is limited cross-protection to intestinal infection between Group B and C serovars of *S. enterica*, suggesting that effective multivalent vaccines for use in controlling *Salmonella* in poultry will be difficult to achieve. However, both the study by Beal et al. (60) and a recent vaccination study have demonstrated a degree of cross-protection between serogroups B and D (60, 61). The use of the live attenuated *S*. Enteritidis vaccine, Gallivac^®^ Se, alone or in combination with the *S*. Enteritidis–*S*. Typhimurium inactivated vaccine, Gallimune^®^ Se + St, prior to infection with either *S*. Typhimurium or *S*. Enteritidis, resulted in a significant reduction in liver and cecal content colonization in the vaccinated compared to unvaccinated chickens (61).

Overall, the findings in this study have shown that *S*. Virchow colonizes chickens and stimulates an inflammatory immune response, similar to that found with broad-range serovars. *S*. Virchow colonized the ileum of chickens to high levels and caused transient systemic infection. The chickens exhibited no clinical symptoms and this, in combination with the only mild histological changes indicates *S*. Virchow has low pathogenicity in chickens. Although the present study has shown that *S*. Virchow has similar infection biology to broad-range serovars, it is rarely isolated from sources other than humans and chickens (13, 62). In contrast, *S*. Typhimurium is commonly isolated from many species including humans, chickens, pigs, cattle, mice, and domestic animals (63). Although *S*. Virchow could cause disease in these hosts and showed similar infection biology to *S*. Typhimurium, it is therefore, likely to be more host-adapted than *S*. Typhimurium.

*Salmonella* Virchow stimulated an acute inflammatory response in chickens, including a rapid increase in IL-1β, IL-6, and CXCLi2 transcription. Evidence of an IFN-γ T cell mediated response was also apparent. An increase in IFN-γ and a lack of IL-4 suggests the immune response against *S*. Virchow is primarily Th1-mediated, although a strong antibody response is elicited by *S*. Virchow. However, unlike *S*. Typhimurium or *S*. Enteritidis, primary infection with *S*. Virchow offers only limited protection against homologous re-challenge with *S*. Virchow or heterologous challenge with *S*. Typhimurium. This suggests developing effective vaccines to S. Virchow or multivalent cross-serogroup vaccines in the chicken may prove problematical. Nevertheless, the findings in this study present valuable information showing the immune responses produced by chickens against *S*. Virchow and could be built on to enable immunological preventative or therapeutic approaches against the serovar.

## Conflict of Interest Statement

The authors declare that the research was conducted in the absence of any commercial or financial relationships that could be construed as a potential conflict of interest.

## References

[1] Braden CR. *Salmonella enterica* serotype enteritidis and eggs: a national epidemic in the United States. Clin Infect Dis (2006) 43:512–7. 10.1086/505973 16838242

[2] Little CL Surman-Lee S Greenwood M Bolton FJ Elson R Mitchell RT Public health investigations of *Salmonella enteritidis* in catering raw shell eggs, 2002-2004. Lett Appl Microbiol (2007) 44:595–601. 10.1111/j.1472-765X.2007.02131.x 17576219

[3] FSA. Report for the UK survey of *Campylobacter* and *Salmonella* contamination of fresh chicken at retail sale. Food Stand Agency (2009).

[4] EFSA. The European Union summary report on trends and sources of zoonoses, zoonotic agents and food-bourne outbreaks in 2010. EFSA J (2012) 10:2597 10.2903/j.efsa.2012.2597 22433599

[5] Ispahani P Slack RCB. Enteric fever and other extraintestinal salmonellosis in University Hospital, Nottingham, UK, between 1980 and 1997. Eur J Clin Microbiol Infect Dis (2000) 19:679–87. 10.1007/s100960000341 11057501

[6] Matheson N Kingsley RA Sturgess K Aliyu SH Wain J Dougan G Ten years experience of *Salmonella* infections in Cambridge, UK. J Infect (2010) 60:21–5. 10.1016/j.jinf.2009.09.016 19819256

[7] Gulcan A Bayram P Levent B Gulcan E. A case of urinary tract infection due to *Salmonella enterica* serovar Virchow and review of the related literature. Acta Microbiol Immunol Hung (2012) 59:85–9. 10.1556/AMicr.59.2012.1.9 22510290

[8] Snow LC Davies RH Christiansen KH Carrique-Mas JJ Wales AD O’Connor JL Survey of the prevalence of *Salmonella* species on commercial laying farms in the United Kingdom. Vet Rec (2007) 161:471–6. 10.1136/vr.161.14.471 17921438

[9] Snow LC Davies RH Christiansen KH Carrique-Mas JJ Cook AJ Teale CJ Survey of the prevalence of *Salmonella* on commercial broiler farms in the United Kingdom, 2005/06. Vet Rec (2008) 163:649–54. 10.1136/vr.163.22.649 19043089

[10] Arnold ME Papadopoulou C Davies RH Carrique-Mas JJ Evans SJ Hoinville LJ. Estimation of *Salmonella* prevalence in UK egg-laying holdings. Prev Vet Med (2010) 94:306–9. 10.1016/j.prevetmed.2010.01.004 20116871

[11] Weinberger M Keller N. Recent trends in the epidemiology of non-typhoid *Salmonella* and antimicrobial resistance: the Israeli experience and worldwide review. Curr Opin Infect Dis (2005) 18:513–21. 10.1097/01.qco.0000186851.33844.b2 16258325

[12] Weinberger M Solnik-Isaac H Shachar D Reisfeld A Valinsky L Andorn N *Salmonella enterica* serotype Virchow: epidemiology, resistance patterns and molecular characterisation of an invasive *Salmonella* serotype in Israel. Clin Microbiol Infect (2006) 12:999–1005. 10.1111/j.1469-0691.2006.01466.x 16961637

[13] Bonalli M Stephan R Kappeli U Cernela N Adank L Hachler H. *Salmonella enterica* serotype Virchow associated with human infections in Switzerland: 2004-2009. BMC Infect Dis (2011) 11:49. 10.1186/1471-2334-11-49 21345197 PMC3050727

[14] Bachmann NL Petty NK Ben Zakour NL Szubert JM Savill J Beatson SA. Genome analysis and CRISPR typing of *Salmonella enterica* serovar Virchow. BMC Genomics (2014) 15:389. 10.1186/1471-2164-15-389 24885207 PMC4042001

[15] Jones TF Ingram LA Cieslak PR Vugia DJ Tobin-D’Angelo M Hurd S Salmonellosis outcomes differ substantially by serotype. J Infect Dis (2008) 198:109–14. 10.1086/588823 18462137

[16] Bertrand S Weill FX Cloeckaert A Vrints M Mairiaux E Praud K Clonal emergence of extended-spectrum beta-lactamase (CTX-M-2)-producing *Salmonella enterica* serovar Virchow isolates with reduced susceptibilities to ciprofloxacin among poultry and humans in Belgium and France (2000 to 2003). J Clin Microbiol (2006) 44:2897–903. 10.1128/JCM.02549-05 16891509 PMC1594617

[17] Hopkins KL Batchelor MJ Anjum M Davies RH Threlfall EJ. Comparison of antimicrobial resistance genes in nontyphoidal salmonellae of serotypes enteritidis, Hadar, and virchow from humans and food-producing animals in England and Wales. Microb Drug Resist (2007) 13:281–8. 10.1089/mdr.2007.779 18184054

[18] Solnik-Isaac H Weinberger M Tabak M Ben-David A Shachar D Yaron S. Quinolone resistance of *Salmonella enterica* serovar Virchow isolates from humans and poultry in Israel: evidence for clonal expansion. J Clin Microbiol (2007) 45:2575–9. 10.1128/JCM.00062-07 17596371 PMC1951243

[19] Meakins S Fisher IS Berghold C Gerner-Smidt P Tschape H Cormican M Antimicrobial drug resistance in human nontyphoidal *Salmonella* isolates in Europe 2000-2004: a report from the Enter-net International Surveillance Network. Microb Drug Resist (2008) 14:31–5. 10.1089/mdr.2008.0777 18366323

[20] Chu C Doublet B Lee YL Cloeckaert A Chiou CS Chen SW *Salmonella* genomic island 1-J variants associated with change in the antibiotic resistance gene cluster in multidrug-resistant *Salmonella enterica* serovar Virchow isolated from humans, Taiwan, 2004-2006. Clin Microbiol Infect (2012) 18:47–53. 10.1111/j.1469-0691.2011.03464.x 21615827

[21] Barua H Biswas PK Olsen KE Shil SK Christensen JP. Molecular characterization of motile serovars of *Salmonella enterica* from breeder and commercial broiler poultry farms in Bangladesh. PLoS One (2013) 8:e57811. 10.1371/journal.pone.0057811 23483931 PMC3590279

[22] Barua H Biswas PK Talukder KA Olsen KE Christensen JP. Poultry as a possible source of non-typhoidal *Salmonella enterica* serovars in humans in Bangladesh. Vet Microbiol (2014) 168:372–80. 10.1016/j.vetmic.2013.11.020 24355536

[23] Gantois I Eeckhaut V Pasmans F Haesebrouck F Ducatelle R Van Immerseel F A comparative study on the pathogenesis of egg contamination by different serotypes of *Salmonella*. Avian Pathol (2008) 37:399–406. 10.1080/03079450802216611 18622856

[24] Lublin A Sela S. The impact of temperature during the storage of table eggs on the viability of *Salmonella enterica* serovars enteritidis and Virchow in the eggs. Poult Sci (2008) 87:2208–14. 10.3382/ps.2008-00153 18931169

[25] Salisbury AM Bronowski C Wigley P. *Salmonella* Virchow isolates from human and avian origins in England – molecular characterization and infection of epithelial cells and poultry. J Appl Microbiol (2011) 111:1505–14. 10.1111/j.1365-2672.2011.05152.x 21920003

[26] Smith HW Tucker JF. The effect of antibiotic therapy on the faecal excretion of *Salmonella* Typhimurium by experimentally infected chickens. J Hyg (Lond) (1975) 75:275–92. 10.1017/S0022172400047318 1100714 PMC2130296

[27] Barrow PA Huggins MB Lovell MA Simpson JM. Observations on the pathogenesis of experimental *Salmonella* Typhimurium infection in chickens. Res Vet Sci (1987) 42:194–9. 3296063

[28] Beal RK Powers C Wigley P Barrow PA Smith AL Temporal dynamics of the cellular, humoral and cytokine responses in chickens during primary and secondary infection with *Salmonella enterica* serovar Typhimurium. Avian Pathol (2004) 33:25–33. 10.1080/03079450310001636282 14681065

[29] Withanage GS Kaiser P Wigley P Powers C Mastroeni P Brooks H Rapid expression of chemokines and proinflammatory cytokines in newly hatched chickens infected with *Salmonella enterica* serovar Typhimurium. Infect Immun (2004) 72:2152–9. 10.1128/IAI.72.4.2152-2159.2004 15039338 PMC375210

[30] Withanage GS Wigley P Kaiser P Mastroeni P Brooks H Powers C Cytokine and chemokine responses associated with clearance of a primary *Salmonella enterica* serovar Typhimurium infection in the chicken and in protective immunity to rechallenge. Infect Immun (2005) 73:5173–82. 10.1128/IAI.73.8.5173-5182.2005 16041035 PMC1201213

[31] Mast J Goddeeris BM Peeters K Vandesande F Berghman LR. Characterisation of chicken monocytes, macrophages and interdigitating cells by the monoclonal antibody KUL01. Vet Immunol Immunopathol (1998) 61:343–57. 10.1016/S0165-2427(97)00152-9 9613446

[32] Kaiser P Rothwell L Galyov EE Barrow PA Burnside J Wigley P. Differential cytokine expression in avian cells in response to invasion by *Salmonella* Typhimurium, *Salmonella enteritidis* and *Salmonella gallinarum*. Microbiology (2000) 146(Pt 12):3217–26. 11101679 10.1099/00221287-146-12-3217

[33] Setta A Barrow PA Kaiser P Jones MA. Immune dynamics following infection of avian macrophages and epithelial cells with typhoidal and non-typhoidal *Salmonella enterica* serovars; bacterial invasion and persistence, nitric oxide and oxygen production, differential host gene expression, NF-kappaB signalling and cell cytotoxicity. Vet Immunol Immunopathol (2012) 146:212–24. 10.1016/j.vetimm.2012.03.008 22475571

[34] Livak KJ Schmittgen TD. Analysis of relative gene expression data using real-time quantitative PCR and the 2(-Delta Delta C(T)) Method. Methods (2001) 25:402–8. 10.1006/meth.2001.1262 11846609

[35] Weinberger M Andorn N Agmon V Cohen D Shohat T Pitlik SD. Blood invasiveness of *Salmonella enterica* as a function of age and serotype. Epidemiol Infect (2004) 132:1023–8. 10.1017/S0950268803001109 15635958 PMC2870192

[36] Kingsley RA Msefula CL Thomson NR Kariuki S Holt KE Gordon MA Epidemic multiple drug resistant *Salmonella* Typhimurium causing invasive disease in sub-Saharan Africa have a distinct genotype. Genome Res (2009) 19:2279–87. 10.1101/gr.091017.109 19901036 PMC2792184

[37] Schifferdecker B Merchan JA Ahmar C Worthington M Griben A Schainfeld RM Endovascular treatment of septic thrombophlebitis: a case report of a rare complication and review of the literature. Vasc Med (2009) 14:47–50. 10.1177/1358863X08096517 19144779

[38] Eckerle I Zimmermann S Kapaun A Junghanss T. *Salmonella enterica* serovar Virchow bacteremia presenting as typhoid-like illness in an immunocompetent patient. J Clin Microbiol (2010) 48:2643–4. 10.1128/JCM.00217-10 20463163 PMC2897497

[39] Hughes LA Wigley P Bennett M Chantrey J Williams N. Multi-locus sequence typing of *Salmonella enterica* serovar Typhimurium isolates from wild birds in northern England suggests host-adapted strain. Lett Appl Microbiol (2010) 51:477–9. 10.1111/j.1472-765X.2010.02918.x 20809923

[40] Henderson SC Bounous DI Lee MD. Early events in the pathogenesis of avian salmonellosis. Infect Immun (1999) 67:3580–6. 10377142 10.1128/iai.67.7.3580-3586.1999 PMC116547

[41] Kogut MH McGruder ED Hargis BM Corrier DE Deloach JR. Characterization of the pattern of inflammatory cell influx in chicks following the intraperitoneal administration of live *Salmonella enteritidis* and *Salmonella enteritidis*-immune lymphokines. Poult Sci (1995) 74:8–17. 10.3382/ps.0740008 7899216

[42] van Dijk A Tersteeg-Zijderveld MH Tjeerdsma-van Bokhoven JL Jansman AJ Veldhuizen EJ Haagsman HP. Chicken heterophils are recruited to the site of *Salmonella* infection and release antibacterial mature cathelicidin-2 upon stimulation with LPS. Mol Immunol (2009) 46:1517–26. 10.1016/j.molimm.2008.12.015 19187966

[43] Setta AM Barrow PA Kaiser P Jones MA. Early immune dynamics following infection with *Salmonella enterica* serovars enteritidis, infantis, pullorum and gallinarum: cytokine and chemokine gene expression profile and cellular changes of chicken cecal tonsils. Comp Immunol Microbiol Infect Dis (2012) 35:397–410. 10.1016/j.cimid.2012.03.004 22512820

[44] Varmuzova K Matulova ME Sebkova A Sekelova Z Havlickova H Sisak F The early innate response of chickens to *Salmonella enterica* is dependent on the presence of O-antigen but not on serovar classification. PLoS One (2014) 9:e96116. 10.1371/journal.pone.0096116 24763249 PMC3999269

[45] Asheg A Levkut M Revajova V Sevcikova Z Kolodzieyski L Pistl J. T lymphocyte subpopulations and B lymphocyte cells in caecum and spleen of chicks infected with *Salmonella enteritidis*. Acta Histochem (2002) 104:435–9. 10.1078/0065-1281-00666 12553717

[46] Beal RK Powers C Wigley P Barrow PA Kaiser P Smith AL. A strong antigen-specific T-cell response is associated with age and genetically dependent resistance to avian enteric salmonellosis. Infect Immun (2005) 73:7509–16. 10.1128/IAI.73.11.7509-7516.2005 16239553 PMC1273861

[47] Berndt A Wilhelm A Jugert C Pieper J Sachse K Methner U. Chicken cecum immune response to *Salmonella enterica* serovars of different levels of invasiveness. Infect Immun (2007) 75:5993–6007. 10.1128/IAI.00695-07 17709416 PMC2168364

[48] van Hemert S Hoekman AJ Smits MA Rebel JM. Immunological and gene expression responses to a *Salmonella* infection in the chicken intestine. Vet Res (2007) 38:51–63. 10.1051/vetres:2006048 17156737

[49] Pieper J Methner U Berndt A. Characterization of avian gammadelta T-cell subsets after *Salmonella enterica* serovar Typhimurium infection of chicks. Infect Immun (2011) 79:822–9. 10.1128/IAI.00788-10 21078853 PMC3028855

[50] Sasai K Aita M Lillehoj HS Miyamoto T Fukata T Baba E. Dynamics of lymphocyte subpopulation changes in the cecal tonsils of chickens infected with *Salmonella enteritidis*. Vet Microbiol (2000) 74:345–51. 10.1016/S0378-1135(00)00193-0 10831856

[51] Berndt A Pieper J Methner U. Circulating gamma delta T cells in response to *Salmonella enterica* serovar enteritidis exposure in chickens. Infect Immun (2006) 74:3967–78. 10.1128/IAI.01128-05 16790770 PMC1489728

[52] Martins-Green M. The chicken chemotactic and angiogenic factor (cCAF), a CXC chemokine. Int J Biochem Cell Biol (2001) 33:427–32. 10.1016/S1357-2725(01)00029-2 11312111

[53] Kogut MH Rothwell L Kaiser P. Differential regulation of cytokine gene expression by avian heterophils during receptor-mediated phagocytosis of opsonized and nonopsonized *Salmonella enteritidis*. J Interferon Cytokine Res (2003) 23:319–27. 10.1089/107999003766628160 12859858

[54] Kogut MH He H Kaiser P. Lipopolysaccharide binding protein/CD14/TLR4-dependent recognition of *Salmonella* LPS induces the functional activation of chicken heterophils and up-regulation of pro-inflammatory cytokine and chemokine gene expression in these cells. Anim Biotechnol (2005) 16:165–81. 10.1080/10495390500264896 16335810

[55] Chappell L Kaiser P Barrow P Jones MA Johnston C Wigley P. The immunobiology of avian systemic salmonellosis. Vet Immunol Immunopathol (2009) 128:53–9. 10.1016/j.vetimm.2008.10.295 19070366

[56] He H Genovese KJ Kogut MH. Modulation of chicken macrophage effector function by T(H)1/T(H)2 cytokines. Cytokine (2011) 53:363–9. 10.1016/j.cyto.2010.12.009 21208811

[57] Avery S Rothwell L Degen WD Schijns VE Young J Kaufman J Characterization of the first nonmammalian T2 cytokine gene cluster: the cluster contains functional single-copy genes for IL-3, IL-4, IL-13, and GM-CSF, a gene for IL-5 that appears to be a pseudogene, and a gene encoding another cytokine like transcript, KK34. J Interferon Cytokine Res (2004) 24:600–10. 15626157 10.1089/jir.2004.24.600

[58] Desmidt M Ducatelle R Mast J Goddeeris BM Kaspers B Haesebrouck F. Role of the humoral immune system in *Salmonella enteritidis* phage type four infection in chickens. Vet Immunol Immunopathol (1998) 63:355–67. 10.1016/S0165-2427(98)00112-3 9656424

[59] Beal RK Powers C Davison TF Barrow PA Smith AL. Clearance of enteric *Salmonella enterica* serovar Typhimurium in chickens is independent of B-cell function. Infect Immun (2006) 74:1442–4. 10.1128/IAI.74.2.1442-1444.2006 16428801 PMC1360334

[60] Beal RK Wigley P Powers C Barrow PA Smith AL. Cross-reactive cellular and humoral immune responses to *Salmonella enterica* serovars Typhimurium and *Enteritidis* are associated with protection to heterologous re-challenge. Vet Immunol Immunopathol (2006) 114:84–93. 10.1016/j.vetimm.2006.07.011 16935350

[61] Springer S Lindner T Ahrens M Woitow G Prandini F Selbitz HJ Duration of immunity induced in chickens by an attenuated live *Salmonella enteritidis* vaccine and an inactivated *Salmonella enteritidis/*Typhimurium vaccine. Berl Munch Tierarztl Wochenschr (2011) 124:89–93. 10.2376/0005-9366-124-89 21462861

[62] Fashae K Ogunsola F Aarestrup FM Hendriksen RS. Antimicrobial susceptibility and serovars of *Salmonella* from chickens and humans in Ibadan, Nigeria. J Infect Dev Ctries (2010) 4:484–94. 10.3855/jidc.909 20818100

[63] DEFRA *Annual Zoonoses Report UK* DEFRA (2010).

